# A Design of Three-Dimensional Spatial Path Planning Algorithm Based on Vector Field Histogram*

**DOI:** 10.3390/s24175647

**Published:** 2024-08-30

**Authors:** Chenning Zong, Qiaoling Du, Jianxv Chen, Yiran Shan, Yanpei Wu, Zhida Sha

**Affiliations:** College of Electronic Science and Engineering, Jilin University, Changchun 130012, China; zongcn1922@mails.jlu.edu.cn (C.Z.); jxchen1922@mails.jlu.edu.cn (J.C.); shanyr1922@mails.jlu.edu.cn (Y.S.); wuyp1922@mails.jlu.edu.cn (Y.W.); shazd1922@mails.jlu.edu.cn (Z.S.)

**Keywords:** path planning, VFH*, three-dimensional path, robot travel

## Abstract

In this paper, we present a novel three-dimensional spatial path planning algorithm based on the Vector Field Histogram* (VFH*) approach, specifically tailored for underwater robotics applications. Our method leverages the strengths of VFH* in obstacle avoidance while enhancing its capability to handle complex three-dimensional environments. Through extensive simulations, we demonstrate the superior performance of our algorithm compared to traditional methods, such as RS-RRT algorithm. Our results show significant improvements in terms of computational efficiency and path optimality, making it a viable solution for real-time path planning in dynamic underwater environments.

## 1. Introduction

In recent years, human exploration of nature has deepened significantly, leading to the widespread use of robots across various operational fields. Among these applications, research on autonomous navigation and path planning for robots has remained a focal point in robotic development. As research progresses and application areas expand, traditional two-dimensional path planning has become inadequate for meeting diverse application scenarios. Consequently, three-dimensional space path planning has emerged as a prominent direction in robot path planning research [1,2,3].

Three-dimensional path planning is a critical component in the field of underwater robotics, where the environment is inherently more complex and unpredictable compared to terrestrial settings. Traditional two-dimensional planning algorithms are insufficient for such applications due to their inability to account for the vertical dimension, which is crucial in underwater navigation. In recent years, the development of three-dimensional path planning algorithms has garnered significant attention, with various approaches being proposed to tackle the unique challenges posed by underwater environments.

From an information exploration standpoint, robot path planning can traditionally be categorized into two types: global path planning and local path planning. The fundamental distinction between these categories lies in the degree of environmental perception. The former is generally applicable to static environments where all environmental information is known beforehand, whereas the latter is suited for dynamic environments where only partial environmental information can be acquired in real-time [2]. The application scenario for the three-dimensional space path planning algorithm based on VFH* described in this paper pertains to dynamic path planning in an unknown global map. This scenario implies that the local map stored in the robot’s decision layer will be updated as the robot’s position changes, and only partial environmental information can be obtained at any given time.

**Figure 1:**
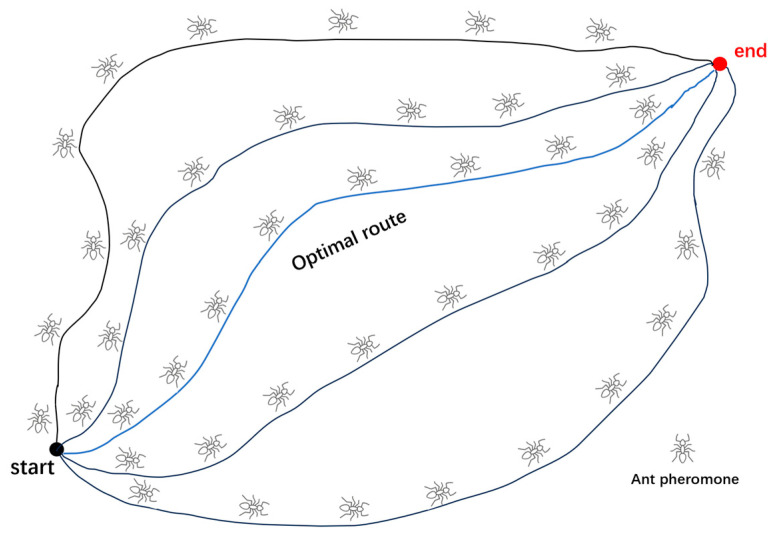
Ant colony algorithm theory.

Global path planning primarily includes algorithms such as the Ant Colony Optimization algorithm [4], A* algorithm [5], and Dijkstra algorithm. Among these, the ant colony algorithm typically employs parallel computing methods, processing map information based on individual population units [6]. The algorithm theory is shown in Figure 1. However, due to its relatively random selection of nodes, the convergence speed of the path is slow in larger travel areas. Additionally, the ant colony algorithm demands high map completeness and is unsuitable for three-dimensional path planning with incomplete map information. Cheng et al. employed the Analytic Hierarchy Process to fit information into weight factors and introduced direction guidance and dynamic optimization, enabling the ant colony algorithm to perform dynamic planning [7]. However, its application was confined to topological maps and could not be generalized to three-dimensional path planning under typical conditions. Similarly, the A* algorithm necessitates pre-processing the map into a mesh node graph and then performing heuristic processing through a predefined cost function. Although heuristic algorithms are straightforward to implement, they are prone to getting trapped in local optima when handling multi-node computations, failing to meet the diverse computational needs of resource migration [8]. Hong ZH et al. designed an improved A* path planning algorithm based on terrain data, utilizing a terrain data map generated from a digital elevation model and devising an optimized search strategy to enhance path planning efficiency [9].

Global path planning typically yields globally optimal paths, but it has a high dependency on the map’s accuracy and completeness. In contrast, local path planning algorithms, such as the DWA (Dynamic Window Approach), RS-RRT algorithm [10], and VFH* algorithm [11,12], have the ability to obtain and process map information in real-time. As a result, these algorithms are more widely used in path planning applications. Masato Kobayashi and Naoki Motoi propose a Dynamic Window Method with a Virtual Manipulator (DWV), which consists of the Dynamic Window Approach (DWA) and a Virtual Manipulator (VM). This method is capable of generating obstacle-avoidance path candidates that include non-linear and non-circular trajectories, effectively preventing collisions with obstacles. However, it requires repeated generation and selection of paths [13]. Ndidiamaka. Adiuku, Nicolas P. Avdelidis, Gilbert Tang, and Angelos Plastropoulos propose an improved hybrid framework based on the RRT algorithm, integrated with theYOLOv7 object detection model, leveraging sensor information from LiDAR and camera systems. This framework enhances the adaptability and navigation capabilities of mobile robots in complex and dynamic environments, significantly improving their ability to perceive, navigate, and interact with various environmental features. However, the system faces issues with response delays, necessitating improvements in navigation speed and operational efficiency [14]. Nevertheless, the algorithm still encounters difficulties in adapting to rapidly changing obstacles in highly dynamic environments. Zong proposed a region-based sampling RRT algorithm that enables RRT to perform real-time planning in the absence of a known global map, thereby meeting the requirements of local path planning [10]. By improving the traditional RRT algorithm, Dynamic RRT has significantly enhanced the speed of path planning, particularly in complex or dynamic environments, where it demonstrates higher efficiency compared to the traditional RRT algorithm. Additionally, Dynamic RRT can quickly respond to environmental changes, reducing the need for frequent path recalculations. However, this method still faces challenges when dealing with extremely complex or rapidly changing environments, such as issues with computational stability. In high-dimensional spaces or very complex environments, the algorithm’s computational resource consumption may be considerable, necessitating further optimization to reduce its reliance on computational resources [15].

The Vector Field Histogram algorithm, abbreviated as the VFH algorithm, is a real-time motion planning algorithm proposed by Johann Borenstein and Yoram Koren in 1991 [11]. This algorithm uses environmental information to obtain the obstacle intensity value in a certain direction of the robot, which then serves as the criterion for path planning. The VFH series algorithms are characterized by a high integration of environmental information and the ability to meet the requirements of dynamic path planning. However, they still have issues such as high sensitivity to threshold values and a tendency to get trapped in local optima.

Babinec, Andrej proposed an improved method for histogram calculation by incorporating the time factor into the prediction tree calculation [16]. This allows VFH to comprehensively consider dynamic obstacles, thereby expanding the application scenarios for the VFH series algorithms. Additionally, the algorithm introduces a new foresight tree design to address obstacle prediction under dynamic conditions. Zhang et al. proposed three reasonable assumptions to predict occluded environments, enhancing the algorithm’s complexity to detect potential paths [17]. This approach enables the VFH* algorithm to more accurately identify interference items and improve the accuracy of map information. Ma et al. used the VFH algorithm to achieve complete coverage path planning (CCPP) for underwater AUV robots [18].

**Figure 2:**
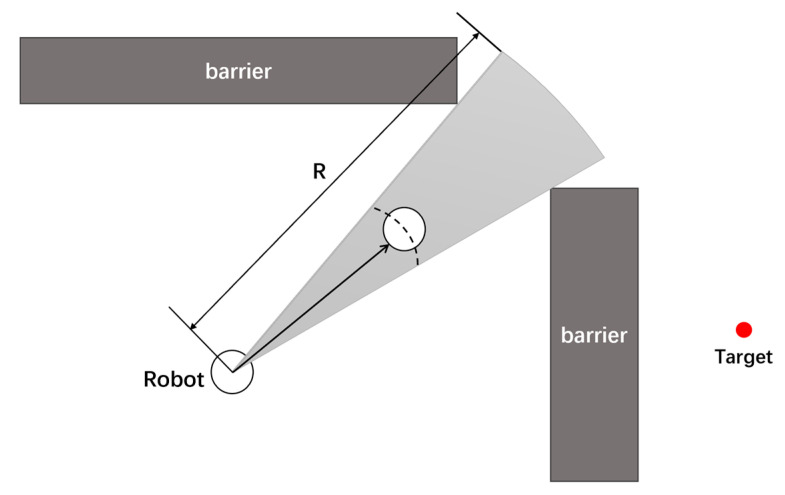
VFH* principle.

The VFH and its derivative algorithms mentioned above can generate effective and smooth obstacle avoidance paths in path planning. Their theory is shown in Figure 2. The research focus lies in the improvement of the algorithm itself. Currently, there are relatively few applications of the VFH algorithm in three-dimensional environments. The sensor’s reading of surrounding environmental information and obstacles is often limited to horizontal and vertical planes, with minimal construction of three-dimensional environments. Their functions are also constrained to two-dimensional planes.

At present, the VFH* algorithm is typically applied to two-dimensional path planning. However, its core mechanism of implementing dual data integrations for complex map information can largely resolve information redundancy in complex environments. Based on the core mechanism of the VFH* algorithm and its subsequent improvements and innovations, this paper designs a three-dimensional space path planning algorithm based on VFH*. Simulation experiments demonstrate that this algorithm can effectively accomplish robot movement and path planning in three-dimensional space, successfully avoiding obstacles in the environment and achieving path optimization for robots in three-dimensional environments.

Our research focuses on enhancing the VFH* algorithm to better suit the requirements of underwater path planning. The original VFH* algorithm, known for its efficiency in obstacle avoidance, operates primarily in two-dimensional spaces. Our goal is to provide an excellent solution for the path planning algorithm of robots in a three-dimensional environment. Our proposed algorithm not only retains the computational efficiency of VFH* but also introduces mechanisms to effectively navigate three-dimensional terrains, accounting for obstacles and varying terrain heights.

## 2. VFH* Algorithm Principle

**Figure 3:**
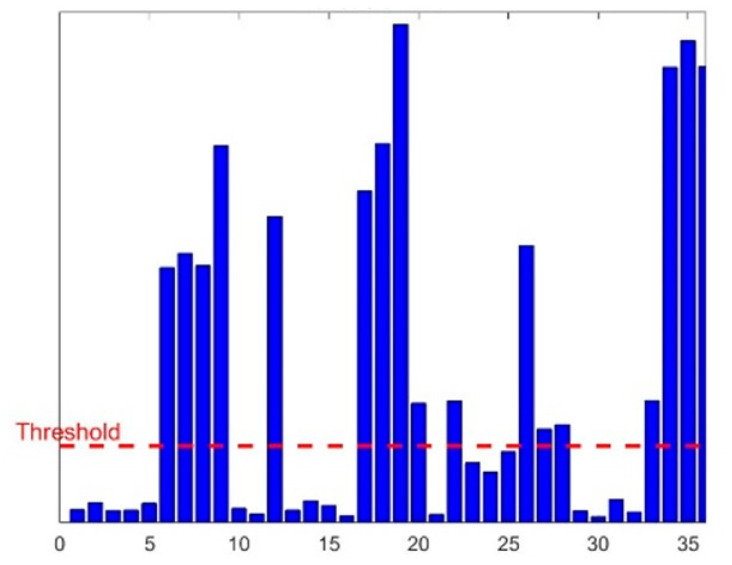
Obstacle cost histogram.

VFH* can be divided into three levels, each level being independent yet interconnected, to collectively complete the algorithm’s work [19]. The highest level is used to describe the robot’s working environment, extract its characteristic value, and summarize them in a grid map. The information at this level is continuously updated and adjusted in real time with the sensors to ensure the timeliness of the information. The middle level is used for data integration processing, quantifying all obstacle information into obstacle strengths ($h_{k}$) at various angles through histogram. To find suitable candidates in the obstacle strength histogram, the algorithm sets a threshold named. It compares each sector with $M_{th}$ and identifies continuous sectors (covering sectors more than $S_{\min}$) that satisfy $h_{k}<M_{th}$ as “candidate valleys”. It then obtains the candidate travel direction from these candidate valleys, as shown in Figure 3.

**Figure 4:**
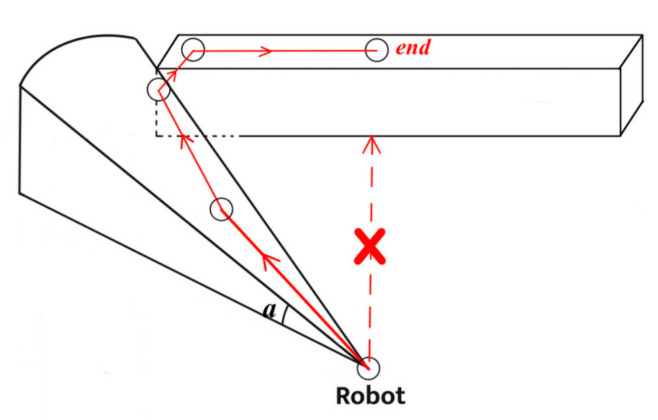
Dead zones that a robot may encounter while traveling.

The bottom level involves predicting nodes established along the candidate direction to address potential dead zones, as shown in Figure 4.

The process of node prediction involves three important parameters: the distance of each exploration ($d_{s}$), the number of explorations ($n_{g}$), and the lookahead distance ($d_{t}$). These three parameters determine the algorithm’s ability to obtain the optimal path and deal with dead zones. The selection of the lookahead distance is generally close to the robot’s radius, and the three parameters satisfy the following relationship [12]:

(1) $$d_{t}=d_{s}\times n_{g}$$

Repeating the above process will yield a predicted node tree based on the robot’s current position, where each node in the tree will generate a corresponding directional cost. The calculation formula is as follows:

(2) $$g(c)=\lambda^{i}\times [\mu_{1}\times \Delta (c_{0},k_{t})+\mu_{2}\times \Delta (c_{0},k_{n})-\mu_{3}\times S]$$

In this formula, $\lambda^{i}$ represents the reduction coefficient of the *i*-th level node (the reduction coefficient of the original node is 0). For the generated prediction tree, use Depth-First Search (DFS) to find the path with the minimum cost as the travel direction.

**Figure 5:**
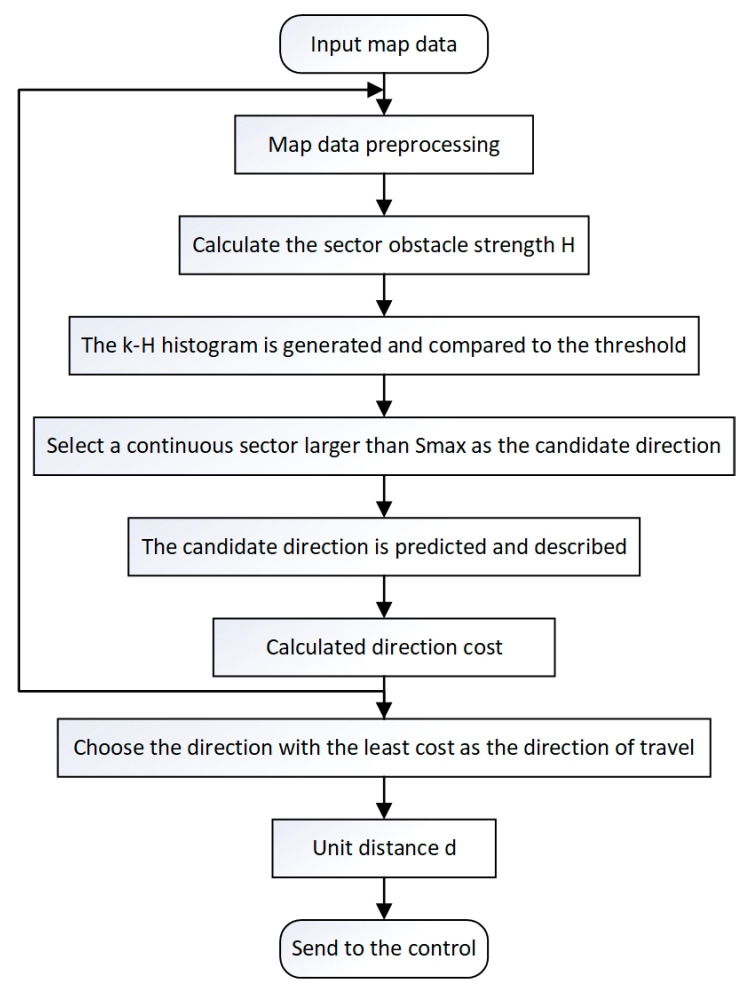
Flow chart of VFH* algorithm.

The flowchart of the VFH* algorithm is shown in Figure 5. The core mechanism of the VFH* algorithm lies in integrating complex map information through two stages [20]:

1. Describing and summarizing the characteristics of environmental information and updating them in real-time, storing complex environmental information in a grid map to complete integration.
2. Calculating obstacle strength in the grid map through a cost function, serving as the evaluation basis for candidate directions.

The VFH* algorithm mentioned above has excellent data integration capabilities in path planning. However, its obstacle strength calculation function is limited to the influence of obstacle distance factors, restricting its functionality to two-dimensional planes and failing to meet the requirements of robots for three-dimensional space path planning in real life. Therefore, to expand the functionality of VFH* to three-dimensional space, it is necessary to improve the integration of information in the local map and the calculation formula for obstacle strength in the sectors. To this end, this paper proposes an integration solution for three-dimensional path information and designs a corresponding obstacle strength calculation formula, aiming to achieve VFH* algorithm in three-dimensional space path planning.

## 3. Improved VFH* Algorithm

Our enhanced VFH* algorithm introduces several key modifications to handle three-dimensional environments effectively. The core idea remains rooted in the original VFH* framework, where a polar histogram is used to represent obstacle densities.

### 3.1. Map Data Preprocessing

When sensors collect a large amount of map information, the primary task is to summarize and preprocess [21]. The essence of preprocessing is to integrate complex terrain information through sensor systems paired with algorithms to extract terrain feature information from the map. In this algorithm, to address the three-dimensional environment, we extract basic information such as the distance (d), the bearing Angle (θ), and confidence value (CV) of obstacles. Additionally, we use sensor arrays to obtain the height values of obstacles (h) and fit their approximate slopes (s). All these obstacle information obtained through preprocessing will undergo a series of fuzzy processing to form obstacle strength, providing criteria for the algorithm.

Before path planning, the environment is discretized into a grid of voxels. Each voxel’s occupancy is determined based on sensor data, which provides information about the presence of obstacles and terrain heights. This preprocessing step is crucial for constructing an accurate three-dimensional cost histogram, which forms the basis of our path planning algorithm.

**Figure 6:**
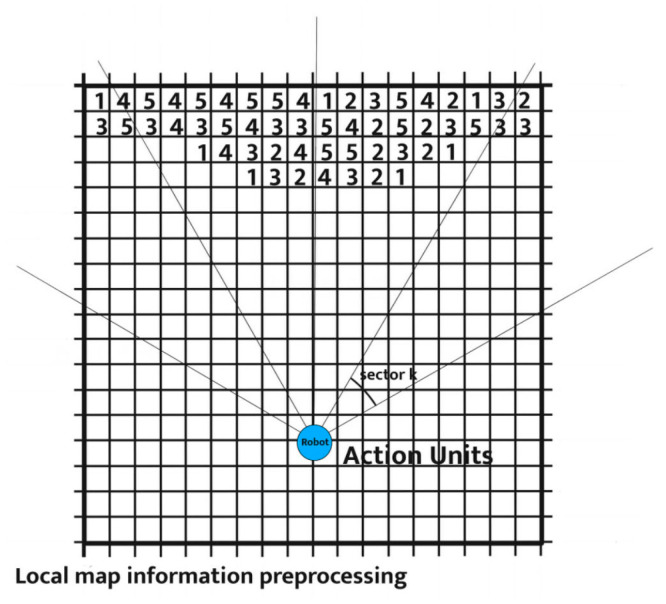
Local map representation.

The VFH* algorithm uses a polar coordinate grid method to store local map information. With the robot’s center position as the origin, the map is converted into grids. Each grid cell has its corresponding polar coordinates, which can be expressed as:

(3) $$I=(l,\theta )\;\;\;(l\in (0,d_{t}),\theta \in (0,2\pi ))$$

where $d_{t}$ represents the maximum exploration distance of the robot. The polar coordinate grid map is divided into sectors, with each sector being labeled in counterclockwise direction and numbered from 1~N. The k-th sector named $S_{k}$ will contain all obstacle points that fall within its angle range. The representation of sectors in the local map is shown in Figure 6.

Under normal circumstances, the range a robot can travel far exceeds its maximum detectable range in a single instance. Therefore, the local map cannot cover the robot’s entire route. To address this issue, we initially select the endpoint and continuously update the surrounding terrain information in real-time during the robot’s journey until the endpoint coordinates appear within the range of the local map.

As the robot moves, the partial map changes with its position. Therefore, updating the partial map is essential. The update formula is given by:

(4) $$\vec{I_{n+1}}=\vec{I_{n}}+\vec{I_{t}}$$

Here, $\vec{I_{t}}$ represents the robot’s displacement, $\vec{I_{n}}$ represents the coordinates of various obstacles in the previous state, and $\vec{I_{n+1}}$ represents the coordinates of obstacles after movement. The partial map will filter the updated coordinates, retaining those that are still within the range of the local map and removing those outside the range. The sensor system will reacquire map information at the new position after movement, and the algorithm will merge identical obstacles, update their confidence $c_{l,\theta }$, and store the newly acquired obstacles in the partial map [22].

Based on this process, the robot can achieve path planning across the global map using the continuously updated local map.

### 3.2. Construction of Cost Histogram

The application scenario for this algorithm is a three-dimensional underwater environment. This environment is characterized by complex and rugged terrain, with significant height differences and predominantly slope-like formations. To address this situation, we have introduced obstacle height (h) and obstacle fit slope (s) to describe the three-dimensional obstacles. Additionally, underwater information is relatively difficult to obtain and subject to high levels of interference. Therefore, we use a fuzzy and integrated approach, processing the multidimensional obstacle information through an obstacle cost function to derive the obstacle strength in a given direction. In summary, we have designed a new cost function in this paper, which is formulated as follows:

(5) $$\left\{\begin{array}{l} m_{l,\theta }={c_{l,\theta }}^{2}\times f_{d}\times f_{h}\times f_{s}\\ f_{d}=a-b\times d_{l,\theta }\\ f_{h}=\left|\ln(-(\beta (s)\times \Delta h+c)\right|+e\\ f_{s}=\left|\ln(-\alpha (s_{t})\times (s-s_{\max}))\right|+n\\ h_{k}=\sum m_{l,\theta } \end{array}\right.$$

Here, $f_{d}$ represents the distance influence factor, and c,e is a constant that satisfies $a-b\times d_{\max}=0$, where $d_{\max}$ represents the farthest distance from the robot’s center point in the grid. This influence factor ensures that the obstacle points at the boundary contributes zero to the obstacle degree. So that the farther away the obstacle point is from the robot, the smaller impact it will have.

$f_{h}$ represents the height difference influence factor, c,e are constants, and β(s) is a weakening function applied to the height difference, satisfying $\beta (s)=0.5+0.6\times s(s\in (0,s_{\max}))$. This influence factor increases with the increase of height difference $h_{\max}$. When there is an obstacle in a sector with a height difference greater than the maximum height the robot can climb, the obstacle strength in this area will reach its maximum value. This factor ensures that excessively high obstacles will significantly affect the obstacle strength of the sector, ensuring that the robot seeks a smoother path for travel.

$f_{s}$ represents the slope influence factor, n is a constant, and satisfying:

(6) $$\alpha =\frac{-1}{s_{t}-s_{\max}}$$

$s_{t}$ is the ideal slope from the current position to the target point, and $s_{\max}$ is the maximum slope the robot can traverse. This factor ensures that obstacles with excessively steep slopes will also significantly increase the obstacle strength of the sector. Under the influence of $\alpha (s_{t})$, the algorithm can achieve an adaptive slope selection strategy related to the height difference between the robot and the target point.

In summary, applying the above cost function allows obstacles to be classified into two scenarios. The first scenario involves obstacles with a small height difference compared to the current position, resulting in a relatively gentle slope and a lower directional cost, generally considered as a passable area. The second scenario involves obstacles with a large height difference, resulting in a steep slope and a high directional cost after processing by the cost function, effectively preventing the robot from moving in that direction, generally considered as an impassable area.

The above adjustment achieves the transformation of algorithm adaptability from two-dimensional space to three-dimensional space. The core enhancement points lie in the establishment of the two factors of and, integrating multi-dimensional information into the cost function to acquire the obstacle intensity and form a brand-new cost function. The cost estimation of obstacles is more precise, allowing it to be applicable to the three-dimensional under-water environment.

## 4. Three-Dimensional Path Planning Algorithm Flow Based on VFH*

After the introduction of the map information preprocessing and the improvement of the sector obstacle strength function, the VFH* algorithm has been significantly enhanced. These advancements allow VFH* to effectively classify and filter obstacles within valleys and predict paths in three dimensions. This enables the algorithm to exhibit superior performance in navigating uneven underwater terrain. The specific steps of the algorithm are as follows:

step1: Obtain the current position information of the robot and the target position information.

step2: Preprocess the environmental information to obtain the local map.

step3: Divide the local map into sectors, and calculate the total obstacle strength of each sector.

step4: Set a threshold $h_{k}<M_{th}$ for sector obstacle strength. Traverse all map sectors and compare their obstacle strength with the threshold to find continuous sectors that satisfy the threshold $h_{k}<M_{th}$, denoted as “candidate valleys”.

step5: Classify and process the candidate valleys to obtain their corresponding candidate direction angles. Concatenate all candidate directions.

step6: Traverse all the direction angles and detect forward the distance of $d_{s}$ and mark this node as a prediction node. Calculate the directional cost of the direction vector (g(c)) corresponding to this predicted node.

step7: Iterate the above step (node prediction) $n_{g}$ times. For each predicted node, calculate its directional cost g(c) and multiply $\lambda^{i}$ by the reduction factor corresponding to the iteration times to obtain g′(c) for different prediction layers. Obtain the complete prediction tree based on the current position.

step8: Use Depth-First Search (DFS) to traverse the prediction tree. Calculate the complete path directional cost using the directional cost as the index. Find the minimum path, and get its initial travel direction.

step9: Output the corresponding results. Determine the distance between the current position and the target point. If the distance is less than 0.5, end the algorithm; otherwise, enter a new iteration.

**Figure 7:**
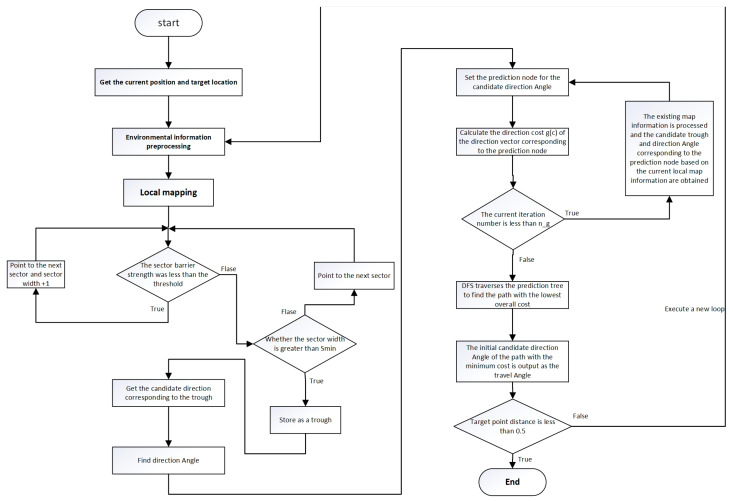
3D path planning algorithm based on VFH*.

The flow chart and pseudo-code of the algorithm are shown in Figure 7 and Algorithm 1.
Algorithm 1: Three-Dimensional Path Planning Algorithm Based on VFH***Input: Local map data, robot parameters, starting point (start), destination point (end)**Obstacle_tree=Obstacle_Init();do{    Obstacle_insert(newdata);    histogram=histogram_create(Obstacle_tree);    while(histogram!=NULL){            sector_start=histogram->k;            if(histogram->cost<threshold){                   while(histogram->next->cost<threshold){                            S++;                   }                   if(S_min<S<S_max){                            angle=AngleOwn(sector_start,S);                   }                   else if(S>S_max){                            angle=target_angle;                   }                   else{                            histogram=histogram->next;                            continue;                   }                   direction_list=Candidate_direction_insert(angle);            }            histogram=histogram->next;    }    forecast_tree=ForecastTree_create( );    while(list!=NULL){            forecast_data=displace(n_g,angle);            cost=cost_calculate(list);            ······            list=list->next;    }    min_record=dfs(forecast_tree);    path_point=Polar_to_Rect(distance,min_record);    heu_distance=dis_calculate(present,end);    path_point_group=dtata_insert(path_point);    Obsatcle_update(path_point );    return path_point;}while (heu_distance <0.5)

## 5. Simulation and Experiment

In order to verify the effectiveness and reliability of the three-dimensional path planning algorithm based on VFH*, simulation experiments are conducted in a simulated terrain environment. The key parameters of the hexapod robot and the three-dimensional path planning algorithm based on VFH* are listed in Table 1. The parameters for the VFH* algorithm, such as the histogram resolution and smoothing factors, are carefully selected based on empirical studies. We also introduce adaptive parameters that adjust in real-time based on the density of obstacles and the complexity of the terrain, enhancing the algorithm’s flexibility and performance.

**Figure 8:**
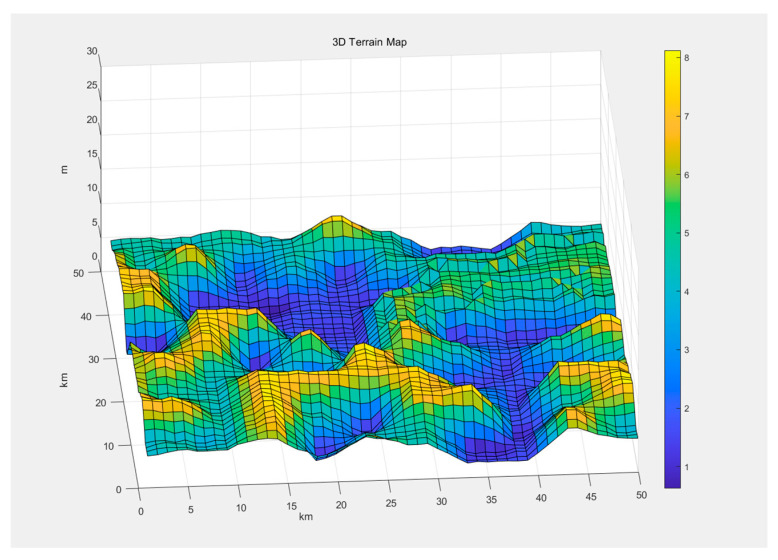
Map information preparation.

To evaluate the performance of our algorithm, we conduct extensive simulations in a variety of underwater environments. Based on the analysis and summary of underwater terrain data, to ensure that the simulated terrain can precisely simulate the actual underwater topography and accurately restore its characteristics, the said simulated terrain is generated in a block random fashion [23]. This approach can accurately replicate the undulating and intricate features of the underwater terrain. The randomly generated nature guarantees the high universality of the terrain data, and its gradient setting ensures the high fidelity of the block distribution [24]. Consequently, this simulated terrain can be applied in the simulation of the genuine underwater terrain and serve as the map substrate for simulation experiments. The map for the simulation experiment is set to be 50 m × 50 m, with heights ranging from 2 dm to 8 dm. The complete map information is shown in Figure 8. The starting point is at (0,0), and the destination is at (40,40), creating a terrain with significant elevation changes between the start and end points.

**Figure 9:**
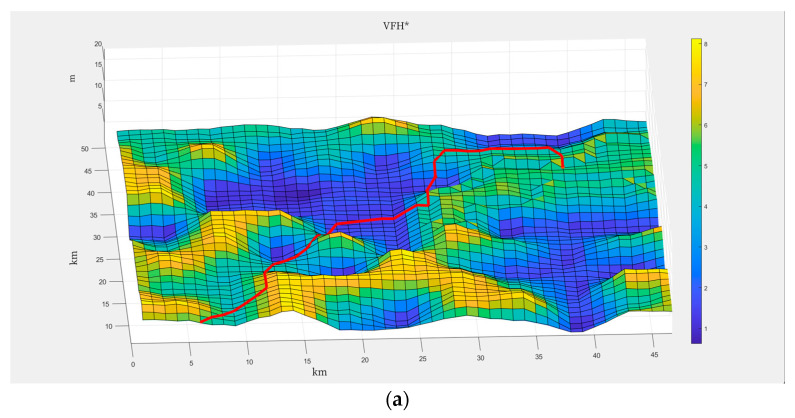

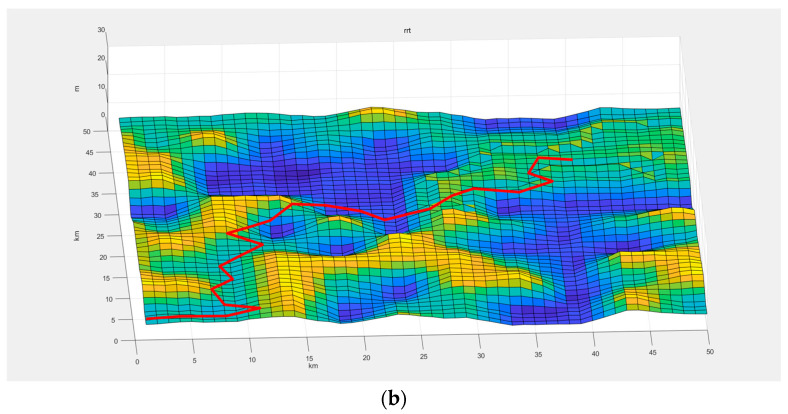
3D path simulation of the two algorithms. (**a**) Simulation results of 3D space path planning algorithm. (**b**) Simulation results of RS-RRT Algorithm.

The algorithm designed in this study will be compared with the regional-Sampling RRT(RS-RRT) algorithm, which refers to the algorithm proposed by Zong [10] and performs a simple three-dimensional terrain adaptation. The path diagram for these two algorithms is shown in Figure 9.

**Figure 10:**
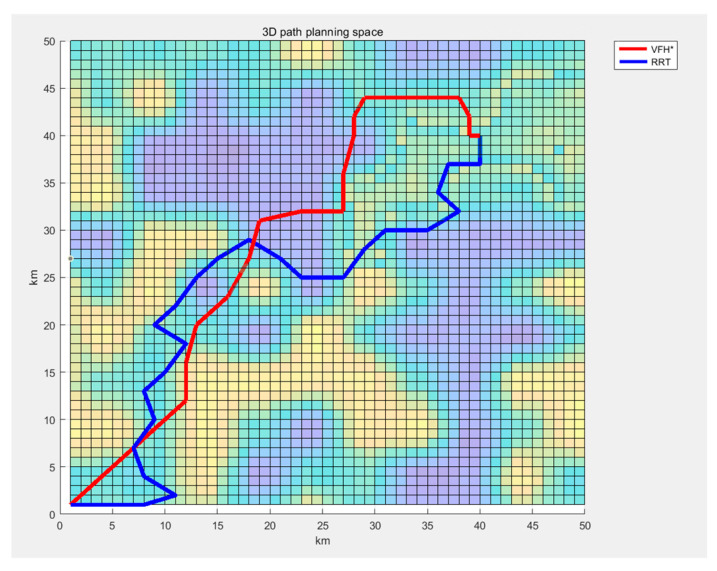
Comparison of two algorithms’ path plan.

Figure 10 shows the comparison between the three-dimensional spatial path planning algorithm based on VFH* and the Region Sampling RRT(RS-RRT) algorithm in terms of flat paths. It can be seen that compared with the RS-RRT algorithm, the proposed algorithm has higher smoothness and less return-route.

The tortuosity of the paths generated by the two algorithms was calculated using the tortuosity formula as shown in Table 2, which satisfies:

(7) $$S=\sum_{i=2}^{n-1}\cos\theta_{i}$$

where $\theta_{i}$ follows:

(8) $$\cos\theta_{i}=\frac{\left(x_{i}-x_{i-1},y_{i}-y_{i-1})*(x_{i+1}-x_{i},y_{i+1}-y_{i}\right)}{\sqrt{{\left(x_{i}-x_{i-1}\right)}^{2}+{\left(y_{i}-y_{i-1}\right)}^{2}}*\sqrt{{\left(x_{i+1}-x_{i}\right)}^{2}+{\left(y_{i+1}-y_{i}\right)}^{2}}}$$

It is evident that the smoothness of the paths generated by the improved VFH* algorithm is significantly higher than that of the Region Sampling RRT(RS-RRT) algorithm. Simultaneously, the evaluation of an algorithm’s performance also includes the overall path length. The path lengths for both algorithms are also included in Table 2.

In contrast, the improved VFH* selects a smoother path within this range. It is evident that the algorithm proposed in this paper is significantly superior to the RS-RRT algorithm in terms of path smoothness.

**Figure 11:**
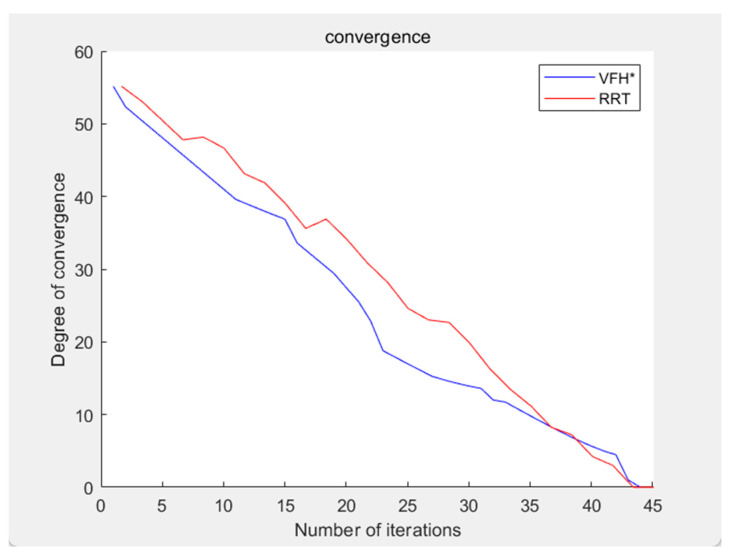
Comparison of convergence rates.

The convergence curves of both algorithms are shown in Figure 11. In this simulation, the algorithm’s convergence can be simply measured by the distance between the coordinates after each move and the specified endpoint coordinates. Let the convergence be denoted as C, which satisfies:

(9) $$C=\sqrt{{\left(x_{e}-x\right)}^{2}{+(y}_{e}-{y)}^{2}+{\left(z_{e}-z\right)}^{2}}$$

From the figure, it can be observed that the improved VFH algorithm demonstrates a higher degree of convergence compared to the RS-RRT algorithm, with a relatively smoother convergence speed. Additionally, it exhibits greater stability when dealing with larger map areas.

**Figure 12:**
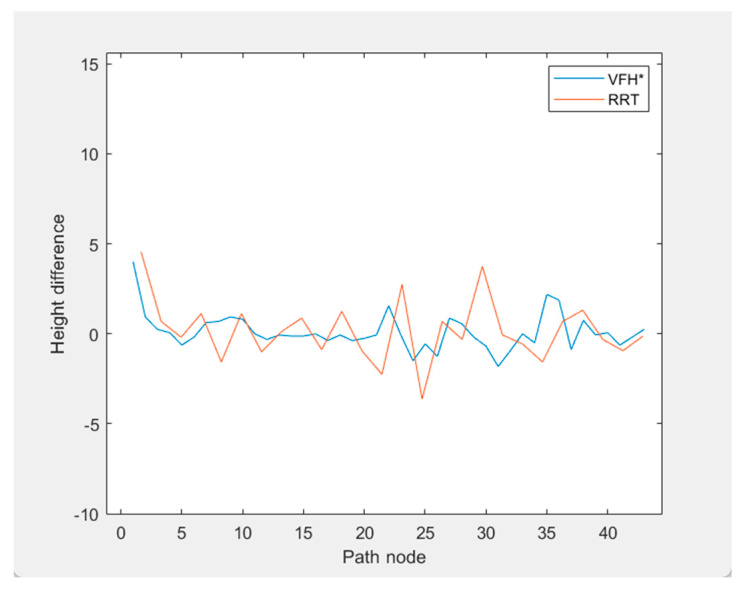
High fluctuation.

Additionally, Figure 12 illustrates the elevation profiles of the paths generated by both algorithms. The improved VFH* algorithm results in smaller elevation changes during ascents and descents compared to the Region Sampling RRT algorithm, leading to smoother ascent paths.

Therefore, the three-dimensional spatial path planning algorithm based on VFH* has a faster convergence rate and stronger performance compared to the RS-RRT algorithm. An algorithm that converges more quickly generally finds the optimal solution in fewer iterations, which translates to higher efficiency. In this comparison, the improved VFH* algorithm demonstrates lower convergence and maintains a consistent convergence rate even after 15 iterations. This consistent and faster convergence indicates that the improved VFH* algorithm is more efficient than the RS-RRT algorithm, as it can reach a better solution in less time, which is particularly important in computationally intensive tasks like three-dimensional spatial path planning.

Time complexity is an important metric for evaluating algorithms. After analysis, the time complexity of the improved VFH* algorithm and the Region Sampling RRT algorithm is determined to between O(NlogN) and $O(N^{2})$. There is no significant difference in time complexity between them.

The comprehensive comparison indicates that the three-dimensional spatial path planning algorithm based on VFH* proposed in this paper can achieve path planning for hexapod robots in underwater three-dimensional terrain. The newly added height difference impact factor and slope impact factor effectively guide the robot to choose the appropriate slope for travel. The prediction tree modeled after VFH* also effectively avoids falling into local extremum situations.

**Figure 13:**
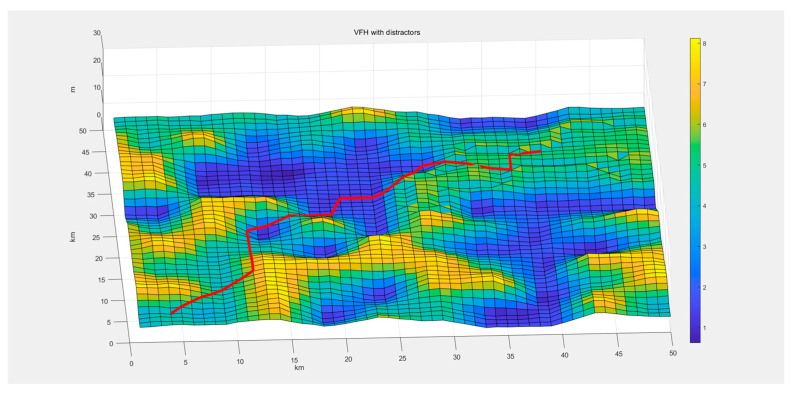
Simulation results of three-dimensional space path planning algorithm with distractors.

To better align with real-world conditions, we introduced system disturbances in the simulation module to test the algorithm’s robustness against external interferences. Gaussian noise was selected to simulate the real environment, following the normal distribution. To mitigate the impact of noise interference, an interval observer was incorporated to minimize the effect of random noise on underwater terrain recognition. The simulated path is shown in Figure 13.

It is evident that even under the simulated noise interference, the algorithm still produces a relatively smooth convergent path, thanks to the calibration effect of the interval observer. The tortuosity of the path is 9.6567.

In summary, its path smoothness, convergence speed, overall path length, and other evaluation criteria are superior to the Region Sampling RRT algorithm in underwater path planning.

## 6. Brief Summary

In the process of robot execution tasks, reasonable three-dimensional obstacle avoidance path planning in complex environments can effectively improve the efficiency and safety of robots. This paper draws on the core mechanism of data integration in the VFH* algorithm to design a three-dimensional spatial path planning algorithm. The algorithm improves the obstacle intensity function, and uses the weighting of the three influence factors $f_{d}$, $f_{s}$, and $f_{h}$ to obtain the overall obstacle intensity, so as to realize the comprehensive consideration of the obstacle height, slope and distance. Through simulation experiments compared with the Region Sampling RRT algorithm, the results show that the algorithm has smoother paths and faster convergence speed.

In conclusion, our enhanced VFH* algorithm provides a robust and efficient solution for three-dimensional path planning in underwater robotics. By extending the VFH* framework to three-dimensional environments and introducing adaptive parameters, we achieve significant improvements in path optimality and computational efficiency. Future work will focus on further refining the algorithm and exploring its application in more diverse underwater scenarios.

In contrast to the traditional VFH series algorithms that correspond to two-dimensional planar path planning, the proposed VFH*-based three-dimensional spatial path planning algorithm broadens the application scenarios of the algorithm. It includes three-dimensional terrains with minor undulations and is suitable for the movement and navigation of multi-legged robots capable of off-road travel.

The premise of the algorithm studied in this paper is that there are no moving obstacles in the environment, which provides a direction for improving future three-dimensional spatial path planning algorithms. Future research will explore the integration of our algorithm with advanced sensor fusion techniques to enhance environmental perception. Additionally, we aim to validate the algorithm in real-world underwater robotic systems, addressing challenges such as sensor noise and dynamic environmental changes.

## Figures and Tables

**Table 1:** Physical parameters in algorithm parameter Settings.

Symbol	Value	Parameter
N	360°	Sensor scan range
n	10°	Angle range per sector
Sn	36	Total number of sectors
a, b	20, 2	Distance factor constant
c, e	1.6, 0.5	Height difference factor
m	0.5	Slope factor constant
$d_{s}$	3	Forward lookahead distance
$n_{g}$	3	Iteration times of lookahead
$d_{t}$	9	Total lookahead distance
D	5	Robot’s travel distance per step
$h_{\max}$	1.6	Maximum height difference
$s_{\max}$	0.7	Maximum slop

**Table 2:** Performance comparison.

Algorithm Type	VFH*	RS-RRT
tortuosity	10.7493	14.8161
path length	83.5913	95.0774

## Data Availability

Data are contained within the article.
